# Active surveillance of adverse drug events in hospitalized patients with pulmonary arterial hypertension based on the global trigger tool

**DOI:** 10.3389/fphar.2025.1533634

**Published:** 2025-08-14

**Authors:** Mengdan Xu, Shunyu Xiong, Guanquan Chen, Guozhi Li, Suzhen He

**Affiliations:** ^1^ School of Pharmacy, Guangdong Pharmaceutical University, Guangzhou, Guangdong, China; ^2^ NMPA Key Laboratory for Technology Research and Evaluation of Pharmacovigilance, Guangzhou, Guangdong, China; ^3^ Guangzhou Pinyi Information Technology Co., Guangzhou, Guangdong, China; ^4^ Department of Pharmacy, Maoming Hospital of Guangzhou University of Chinese Medicine (Maoming Hospital of Traditional Chinese Medicine), Maoming, Guangdong, China; ^5^ Department of Pharmacy, The First Afffliated Hospital of Guangzhou Medical University, Guangzhou, Guangdong, China

**Keywords:** global trigger tool, pulmonary arterial hypertension, adverse drug events, active monitoring, Chinese hospital pharmacovigilance system

## Abstract

**Objective:**

To explore the method of active monitoring of adverse drug events (ADEs) in patients with a rare disease: pulmonary arterial hypertension (PAH).

**Methods:**

First, initial trigger items were extracted and organized through literature-based evidence and drug inserts. Then, the trigger items were refined using expert consultation based on a Delphi method. Second, patients with PAH admitted to a hospital between 1 January 2022 and 1 June 2023 were extracted as study samples. A retrospective case review was conducted to verify the clinical applicability of triggers. We calculated indicators such as sensitivity, specificity, and positive predictive value (PPV), and also observed the ADEs. Risk factors for the development of ADEs in hospitalized patients with PAH were also analyzed.

**Results:**

We extracted and organized 31 initial trigger items. After two rounds of modification through expert consultation, 24 triggers were identified. We converted the trigger items into risk signals to automatically identify cases with possible ADEs with the help of the Chinese Hospital Pharmacovigilance System (CHPS). Manual review of suspected cases revealed that the overall PPV of the triggers was 30.30%, the sensitivity was 98.21%, and the specificity was 72.57%. The prevalence of ADEs detection was 17.57%, which was equivalent to 25.75 ADEs occurrences in 1,000 patient days and 11.84 ADEs occurrences in 1,000 medications. ADEs-involved organs included the blood system, and there was also damage to the gastrointestinal system, hepatic system, and renal system. ADEs severity was categorized into 24 grade D cases, 102 grade E cases, 15 grade F cases, and two grade H cases. The longer the duration of hospitalization, the higher the number of comorbidities, the higher the number of positive triggers, and the higher the likelihood of ADEs.

**Conclusion:**

This study provides a new method for actively identifying ADEs in hospitalized patients with PAH, as well as a reference for research on pharmacovigilance for rare diseases.

## 1 Introduction

The use of any medication carries potential risks. Adverse drug event (ADE) monitoring began in the 1960s with the thalidomide incident. Pregnant women took thalidomide to treat vomiting during pregnancy, but this resulted in tens of thousands of “seal limbed babies” (Routledge, 1998; Fornasier et al., 2018). Since then, drug safety has become a major global concern. The World Health Organization (WHO) defines an ADE as a “harmful, unplanned adverse event that occurs at a therapeutic dose of a drug” (Nebeker et al., 2004).

The most common and simplest method of ADE monitoring is passive monitoring. This involves spontaneous reporting of ADEs by marketing authorization holders and healthcare providers, among others (National Center for Adverse Drug Reaction Monitoring, 2023). Spontaneous reporting is the main source of ADE reports and an important basis for post-marketing safety evaluation of drugs (Montané and Santesmases, 2020). However, passive surveillance has numerous shortcomings, such as incomplete and outdated reporting and many ADEs going undetected (Garcia-Abeijon et al., 2023).

To counteract the inadequacy of passive monitoring, scholars in various countries have explored active monitoring methods. Active monitoring methods for clinical ADEs include the Global Trigger Tool (GTT), Harvard Medical Practice Research, Comparative Safety Indicator Database, direct observation, and case review. The GTT method is considered to have a particularly high efficacy for monitoring (Ad et al., 2020).

The GTT method is an active monitoring tool introduced by the Institute for Healthcare Improvement (IHI) in 2003. It is a method for purposefully identifying potential ADEs in cases using “triggers” as clues (Classen et al., 2011; Hu et al., 2019). In 2009, the IHI released the second edition of the *Global Trigger Tool for Measuring Adverse Events* (GTT White Paper). The latter categorizes triggers into six modules, namely, nursing (C), medications (M), surgery (S), intensive care (I), perinatal (P), and emergency (E), and recommends 51 triggers (Griffin and Resar, 2009). The GTT has been applied widely for more than 2 decades after it was released by the IHI. To conduct active monitoring of ADEs in a more targeted manner, researchers have specified monitoring to specific departments, such as in emergency medicine (Rosen and Mull, 2016; Howard et al., 2017; Spangler et al., 2020), surgery (Pérez Zapata et al., 2015), pediatrics (Liu et al., 2018; Sakuma et al., 2024), and intensive care unit (ICU) (Aikawa et al., 2021; Liston et al., 2023), among other departments. For example, in Sweden, Rutberg et al. (2014) used the GTT method for active monitoring of ADEs in patients in one hospital. The prevalence of detection of ADEs was 20.5%, while only 6.3% of ADEs were reported in the spontaneous reporting system during the same period. In the United States, Takata et al. (2008) developed a specific trigger tool for detecting ADEs in children. In Sweden, Nilsson et al. (2012) conducted a structured review of ICU cases in a medium-sized hospital based on GTT. They reviewed 128 cases and identified 41 ADEs in 25 patients.

GTT studies have focused on certain populations, such as children (Ji et al., 2018; Liu et al., 2020), older people (Hibbert et al., 2016), or populations of patients with certain common diseases. Very few studies have been conducted on the active monitoring of ADEs in patients with rare diseases. Pre-marketing studies of drugs for rare diseases are very limited in size; sometimes, only a few dozen patient volunteers can be recruited for clinical trials, and the duration of the studies is short. Furthermore, information on rare adverse reactions, long-term toxicity, and effects on special populations (e.g., children, pregnant women, older people) is often difficult to obtain in pre-marketing studies (Anzelewicz et al., 2017). In addition, in the treatment of rare diseases, patients are often forced to accept off-label medications whose safety is difficult to confirm because rare medications without corresponding indications are approved for marketing (Kleiber et al., 2021). Therefore, it is important to monitor ADEs in patients with rare diseases.

Taking pulmonary arterial hypertension (PAH) as an example, we aimed to establish a triggering mechanism for ADEs and conduct a clinical-applicability validation test for it. In this way, we explored a method for the active monitoring of ADEs in patients with a potentially severe disease.

## 2 Methods

### 2.1 Establishment of triggers

#### 2.1.1 Selection of target drugs

Based on the in-hospital drug supply catalog and a review of historical cases, we decided to use PAH-targeted drugs as the target drugs for the study. There are eight targeted drugs in use in their premises currently: (1) phosphodiesterase type-5 inhibitors (e.g., Sildenafil, Tadalafil); (2) Endothelin Receptor Antagonists (ERA) (Ambrisentan, Macitentan); (3)soluble guanylate cyclase (sGC) stimulant (Riociguat); (4) prostacyclin IP receptor agonists (Selexipag); and (5) Artificially synthesized prostacyclin analogs (Beraprost, Treprostinil).

#### 2.1.2 Preliminary establishment of trigger items

First, the Web of science, PubMed, and China National Knowledge Infrastructure (CNKI) were searched. The English search terms were as follows: “trigger tool,” “adverse drug event,” “adverse event,” “adverse drug reaction,” “adverse reaction,” “pulmonary hypertension,” and “pulmonary arterial hypertension”. The Chinese search terms were as follows: “global trigger tool,” “adverse event,” “adverse reaction,” and “pulmonary arterial hypertension”. The information and triggers related to adverse events of PAH-targeted drugs were extracted and summarized through evidence-based literature.

Second, based on the 51 triggers recommended in the GTT white paper (Griffin and Resar, 2009), the triggers relevant to the present study were extracted.

Third, we extracted ADE information from drug inserts. The risk signals for ADE occurrence were organized and extracted based on the adverse events, contraindications, and precautions mentioned in the drug inserts of the target drugs Ambrisentan, Macitentan, Sildenafil, Tadalafil, Riociguat, Selexipag, Beraprost, Treprostinil.

Fourth, we extracted adverse-event risk signals from databases focusing on ADE reporting systems. The Chinese Adverse Drug Reaction Feedback Database, the European Database of Suspected Adverse Drug Reaction Reports, and the FDA Adverse Event Reporting System were searched. Then, we extracted the risk signals for ADEs of PAH-targeted drugs reported in these databases.

The trigger entries extracted from the four areas stated above and the list of PAH-targeted ADEs were combined and summarized to form the initial trigger items. Trigger items were categorized into the categories of “Laboratory Indicators (L),” “Antidotes (A),” “Clinical Symptoms (S),” and “Treat (T).”

#### 2.1.3 Delphi method for optimization of trigger items

We designed a Delphi expert consultation questionnaire based on the initial trigger items. The selection criteria for experts are as follows: (1) Possess a bachelor’s degree or above; (2) Clinical doctors or pharmacists who have rich experience in clinical medicine or clinical pharmacy and are engaged in front-line clinical work of pulmonary arterial hypertension. The opinions of experts are quantified through the positive coefficient, authority coefficient (Cr), coefficient of variation (Vj) and coordination coefficient (W) of experts.

Ultimately, we selected 19 experts in clinical medicine and pharmacy from six tertiary grade A hospitals in Guangzhou, Shanghai and Jiangmen for consultation. After two rounds of consultation, the positive coefficient of the experts was 100%, Cr = 0.82 ± 0.03, Vj = 0.16 ± 0.04, and W = 0.365. It indicates that the enthusiasm and authority of the experts are both relatively high, and the differences in their opinions are relatively small, with a relatively high overall coordination.

We determined the final trigger based on the cut-off values of three indicators, namely, the mean importance score (Mj), the frequency of full marks (Kj), and the coefficient of variation (Vj), as well as expert opinions. The calculation method for the cut-off values of Mj and Kj for each trigger is “cut-off value = mean - standard deviation”, and those with scores higher than the cut-off values are selected. The calculation method for the cut-off value of Vj is “cut-off value = mean +standard deviation”, and those with scores lower than the cut-off values are selected. Triggers whose three indicators do not fall within the threshold range can be deleted; otherwise, a decision on whether to discard them should be made after discussion in combination with expert opinions.

### 2.2 Validation of the clinical suitability of triggers

We extracted data of patients with PAH admitted to the First Affiliated Hospital of Guangzhou Medical University (Guangzhou, China) from January 1, 2022, to June 1, 2023, with the help of the Chinese Hospital Pharmacovigilance System (CHPS).

The inclusion criteria for cases were as follows: (1) confirmed diagnosis of PAH; (2) treatment with ambrisentan, macitentan, sildenafil, tadalafil, riociguat, selexipag, beraprost, treprostinil for more than 24 h; (3) age >28 days; (4) hospitalization duration ≥24 h; and (5) hospital admission after 1 January 2022 and hospital discharge before 1 June 2023.

The study protocol was approved by the Ethics Committee of the First Hospital of Guangzhou Medical University (ES-2023-076-02).

#### 2.2.1 Review of suspicious cases

Based on the risk signals set by the triggers, CHPS can automatically identify cases where ADEs are likely to occur. Nevertheless, false-positives can occur, such as abnormalities in the indicators caused by disease progression rather than due to the medication. Such false-positives require a professional reviewer to audit the suspicious case to determine if the patient has experienced an ADE.

The Case Review Team comprised two junior reviewers (one clinical pharmacist and one graduate pharmacy student) and two senior reviewers (two chief pharmacists). All members were trained in ADEs prior to case review. The two junior reviewers reviewed suspicious cases independently to determine whether the trigger result was a true-positive or a false-positive. In the event of a disagreement between the two junior reviewers, the decision of whether an ADE had occurred was made by the two senior reviewers with strict adherence to the “20-min rule” to ensure thorough review of the case and to avoid omission of ADE information. The sex, age, hospitalization duration, and number of comorbidities were recorded.

Evaluation of drug-ADE causality was carried out using the criteria set by the World Health Organization-Uppsala Monitoring Centre (WHO-UMC). The latter categorizes causality into six levels: “definitely related,” “very probably related,” “probably related,” “probably not related,” “to be evaluated,” and “not evaluable.” Injury levels caused by ADEs were classified using the error-grading system developed by the National Coordinating Council for Medication Error Reporting and Prevention (NCC MERP) of the United States, which categorizes ADEs into grades A–I, as detailed in Table 1. Only E–I injuries were included in this study.

**TABLE 1:** Error-grading system set by the NCC MERP.

Grade A	Objective circumstances or events have the potential to cause errors to occur
Grade B	Medication errors occurred but the medications were not given to the patient, or else they were given to the patient but not used
Grade C	A medication error occurred and the patient used the medication, but it did not cause any harm to the patient
Grade D	Medication errors did not impair patients, but monitoring is required
Grade E	Medication errors caused temporary harm to patients and required action
Grade F	Medication errors caused temporary harm to patients, resulting in hospitalization or a prolonged hospital stay
Grade G	Medication errors caused permanent damage to patients
Grade H	The medication error resulted in a life-threatening situation for the patient, requiring life-sustaining measures
Grade I	Medication errors led to patient death

#### 2.2.2 Statistical analyses

Excel 2021 (Microsoft, Redmond, WA, United States) and SPSS 26.0 (IBM, Armonk, NY, United States) were used for data analysis. Descriptive statistics were employed for the basic information of patients. Measurement data are presented as the mean ± standard deviation. Count data are expressed as frequencies and percentages.

The GTT method was evaluated using the positive predictive value (PPV, where PPV = number of true positives/number of positives × 100%), sensitivity, specificity, and prevalence of ADE detection. We calculated the following: number of ADE occurrences in 1,000 patient days (1000-patient-day ADE occurrences = total number of ADE occurrences/total time of each case × 1,000); ADE occurrences in 100 patients (100-patient ADE occurrences = total number of ADE occurrences/total number of cases × 100); ADE occurrences over 1,000 drugs (1000-drug ADE occurrences = total number of ADE occurrences/number of medication types in each case × 100); and ADE prevalence = number of patients who had at least one ADE/total number of cases × 100%. Risk factors for PAH development in patients were analyzed using univariate analysis and multifactorial binary logistic regression. Detection using the GTT method was analyzed in comparison with spontaneous reporting during the same period to verify the validity of the GTT method.

## 3 Results

### 3.1 Creation of trigger items

ADE-related triggers for hospitalized patients with PAH were extracted in a multifaceted manner through evidence-based literature, recommended triggers in the GTT white paper, drug inserts, and the database for the spontaneous reporting of ADEs. Thirty-one initial trigger items were extracted and collated.

After expert consultation, the calculation results of the boundary values are: Mj = 3.693, Kj = 11.848%, Vj = 0.2. Based on the threshold values and expert opinions, we revised the initial trigger items and identified 24 triggers: seven laboratory indicators, six antidotes, eight clinical symptoms, and three interventions.

### 3.2 Validation tests for the clinical suitability of triggers

#### 3.2.1 Basic information of patients

Based on the inclusion criteria, we included 626 inpatient with PAH (245 (39.14%) males and 381 (60.86%) females). The age range was 50.04 ± 18.18 years (range, 4 months to 93 years). The duration of hospitalization was 8.87 ± 8.08 (range, 1–68) days. The number of combined targeted drug classes was 1.93 ± 0.95 (1–6 types). The number of comorbidities was 5.74 ± 3.63 (range, 1–25). The number of previous hospitalizations was 3.65 ± 3.55 (range, 1–24). Respiratory medicine, general pediatrics, rheumatology, and orthopedic surgery were the main specialties involved (Table 2).

**TABLE 2:** Comparison of patients with and without ADEs.

Variant		Aggregate (n = 626)	Without ADEs (n = 514)	With ADEs (n = 112)	P
Age (years)	0–17	26	19	7	0.601
	18–40	186	153	33	
	41–60	211	172	39	
	>60	203	170	33	
Sex	Male	245	201	44	0972
	Female	381	313	68	
Duration of hospitalization (days)	1–10	491	431	60	**0.000**
	11–20	93	61	32	
	21–30	23	12	11	
	>30	19	10	9	
Number of previous hospitalizations	1–3	403	328	75	0.816
	4–6	137	114	23	
	>6	86	72	14	
Number of targeted drug classes combined	1	242	208	34	0.202
	2	238	189	49	
	3	104	85	19	
	>3	42	32	10	
Number of comorbidities	1–3	198	175	23	**0.000**
	4–6	219	181	38	
	7–9	128	104	24	
	>9	81	54	27	
Number of positive triggers	0–1	494	455	39	**0.000**
	2–4	110	52	58	
	≥5	22	7	15	

Note: P < 0.05 indicates that the occurrence of factor ADE may be correlated.

Assessment of patients with and without an ADE using a one-way chi-square test revealed no significant differences in the number of PAH-targeted drug classes combined, number of prior hospitalizations, age, or sex (P > 0.05). However, for hospitalization duration, number of comorbidities, and number of positive triggers, P < 0.05 was noted, suggesting that these may be influential factors in ADE occurrence.

#### 3.2.2 Trigger detection

We undertook the ADE-detection test with the help of CHPS in 626 patients with PAH. All 24 trigger factors yielded positive results in actual application. Among the 626 patients, 375 had positive triggers, resulting in a total of 472 trigger instances, averaging approximately 0.75 instances per patient. A review of positive cases revealed 143 ADEs involving 110 cases, suggesting that some patients had more than one ADE. The overall PPV of the triggers was 30.30% (143/472) (Table 3).

**TABLE 3:** Detection of 24 triggers for ADEs and positive predictive values.

No.	Triggers	Positive triggers	ADEs	PPV	Triggers classification
L1	Absolute eosinophils >0.3 × 10^9^/L (Härkänen et al., 2020)	6	4	66.67%	high utility
L2	Platelet count <50 × 10^9^/L (Kirkendall et al., 2012; Sekijima et al., 2020)	7	3	42.86%	moderate
L3	Leucocyte count <3 × 10^9^/L (Schulson et al., 2021; Garcia-Abeijon et al., 2023; Hibbert et al., 2023)	5	3	60.00%	high utility
L4	Alanine aminotransferase or aspartate transaminase > 3-times the upper limit of normal (ULN), or total bilirubin and alkaline phosphatase both increased by more than 2-times the ULN (Griffey et al., 2019; Hu et al., 2019)	18	7	38.89%	moderate
L5	Blood urea nitrogen or serum creatinine increased by more than 2-times the baseline level (Hommel et al., 2020)	21	8	38.10%	moderate
L6	6 months–5 years hemoglobin (Hb) < 110 g/L, 5–12 years Hb < 115 g/L, 12–15 years Hb < 120 g/L, males older than 15 years Hb < 120 g/L, females older than 15 years Hb < 110 g/L (Brösterhaus et al., 2020)	88	35	39.77%	moderate
L7	Sodium concentration in blood >145 mmol/L (Grossmann et al., 2019)	5	3	60.00%	high utility
A1	Use of hypertensor drugs such as norepinephrine, dobutamine, mesalamine, and ephedrine (Dolci et al., 2020; Aikawa et al., 2021)	27	5	18.52%	low utility
A2	Use of antiemetics such as metoclopramide, tropisetron, dolasetron, palonosetron, ondansetron, fosaprepitant, aprepitant, or phenformin (Kirkendall et al., 2012; Wong et al., 2019)	28	12	42.86%	moderate
A3	Use of antidiarrheal drugs such as montmorillonite powder, Bifidobacterium bifidum tetragonum Live Combined Bifidobacterium and *Lactobacillus* Tablets, Bifidobacterium live bacteria, Loperamide, *Saccharomyces* boulardii, or live *Bacillus* cereus bifidus (Liu et al., 2020)	40	14	35.00%	moderate
A4	Use of hepatoprotective drugs such as glutathione, diammonium glycyrrhizinate, magnesium isoglycyrrhizinate, complex glycyrrhizin, monoammonium cysteine glycyrrhizinate, polyenylphosphatidylcholine, ursodeoxycholic acid and silymarin (Wong et al., 2019; Mevik et al., 2019)	22	6	27.27%	moderate
A5	Use of laxatives or stool-softeners such as glycerine enema, lactulose oral solution, hemp nut soft gels, bisacodyl enteric tablets, or senna (Classen et al., 2011; Hu et al., 2019; Lu et al., 2024)	21	7	33.33%	moderate
A6	Use of loratadine, ebastine, chlorpheniramine maleate, olopatadine, cetirizine, cyproheptadine, promethazine, or diphenhydramine (Kirkendall et al., 2012; Dolci et al., 2020)	42	10	23.81%	moderate
S1	Bleeding (including rectal bleeding, nosebleeds, eye bleeding, intra-abdominal bleeding, hemorrhage, bleeding tendencies, severe bleeding, vomiting blood, gastrointestinal bleeding, subcutaneous bleeding, and urinary-tract bleeding) (Wong et al., 2019; Zhang et al., 2022)	14	1	7.14%	low utility
S2	Nausea, vomiting, abdominal pain, diarrhea, and bloating (Hu et al., 2019)	13	5	38.46%	moderate
S3	(1) Arthralgia/joint pain (Sajith et al., 2021); (2) bone pain; (3) jaw pain; (4) back pain; (5) neck pain; and (6) chest pain (Hommel et al., 2020)	6	2	33.33%	
S4	(1) Flushing, facial flushing, or hot flushes; (2) rash, itching, or hives; (3) eczema (Valkonen et al., 2023)	6	4	66.67%	high utility
S5	Peripheral edema, puffiness, edema, or swelling (including angioedema, lower-extremity edema, fluid retention, retinal edema, and facial edema) (Aikawa et al., 2021)	51	5	9.80%	low utility
S6	Dizziness, headaches (Nilsson et al., 2023)	9	5	55.56%	
S7	(1) Insomnia, anxiety, transient amnesia, depression, drowsiness, vertigo, upright vertigo; (2) fainting, loss of consciousness, unconsciousness, numbness, floating sensation; and (3) neuralgia/neuropathy (Sajith et al., 2021; Garcia-Abeijon et al., 2023)	1	1	100.00%	high utility
S8	Kidney injury, acute kidney injury, oliguria, anuria, acute tubular necrosis, tubular dysfunction, proteinuria, hematuria, or chronic kidney damage (Hibbert et al., 2023)	2	0	0.00%	low utility
T1	Transfer to ICU(Naessens et al., 2010; Grossmann et al., 2019)	3	0	0.00%	low utility
T2	Salvage (Griffey et al., 2019)	6	1	16.67%	low utility
T3	Abrupt cessation of medication (Sekijima et al., 2020; Zhang et al., 2022)	31	2	6.45%	low utility
Total		472	143	30.30%	

#### 3.2.3 Characteristics of ADEs

Among 626 patients with PAH, the triggers detected 143 ADEs in 110 patients, with a prevalence of ADE detection of 17.57% (110/626). According to the WHO-UMC assessment method, 119 cases were rated as “possibly related” (83.22%, 119/143), 19 cases were rated as “very likely related” (13.29%, 19/143), and five cases were rated as “definitely related” (3.50%, 5/143). According to the NCC MERP error-grading system, of the 143 ADEs, 24 (16.78%, 24/143) were “D injuries,” 102 (71.33%, 102/143) were “E injuries,” 15 (10.49%, 15/143) were “F injuries,” 2 (1.40%, 2/143) were “H injuries,” and no “G injuries” or “I injuries” were found. The number of ADEs in 1,000 patient days was calculated to be 25.75 (143/5,554 × 1,000), the number of ADEs in 100 patients was calculated to be 22.84 (143/626 × 100), and the number of ADEs over 1,000 drugs was calculated to be 11.84 (143/1,208 × 100).

ADEs can be categorized into nine major groups in terms of the organs and systems involved. The “hematologic system, bleeding, and coagulation disorders” category was the most prevalent, amounting to 46 cases (32.17%, 46/143). These were followed by “damage to the gastrointestinal system” in 38 cases (26.57%, 38/143) and “damage to the liver and kidneys” in 21 cases (14.69%, 21/143). The remainder were “systemic damage,” “metabolic and nutritional disorders,” “skin and adnexal system,” “damage to the central and peripheral nervous system,” and “damage to the musculoskeletal system” (Table 4).

**TABLE 4:** Organs and systems involved in ADEs.

Organ/system	ADEs	Number	Total cases
Blood system, bleeding and clotting disorders	Anemia	35	46 (32.17%)
	Eosinophilia	4	
	Thrombocytopenia	3	
	Leukopenia	3	
	Hemorrhage	1	
Damage to the gastrointestinal system	Nausea, vomiting, abdominal pain, diarrhea, or bloating	31	38 (26.57%)
	Constipation	7	
Liver and kidney damage	Liver injury	13	21 (14.69%)
	Kidney damage	8	
Systemic damage	Allergic reaction	10	10 (6.99%)
Metabolic and nutritional disorders	Hypotension	5	8 (5.59%)
	Hypernatremia	3	
Other	Peripheral edema/puffiness/edema/swelling	5	8 (5.59%)
	Stopping medication suddenly	2	
	Rescue	1	
Damage to the central and peripheral nervous system	Dizziness, headaches	5	6 (4.20%)
	Insomnia, anxiety, transient amnesia, depression, drowsiness, vertigo, upright vertigo, fainting, disorientation, unconsciousness, numbness, floating sensation, neuralgia, neuropathy	1	
Skin and accessory systems	Flushing, facial flushing, hot flushes, rash, itching, hives, or eczema	4	4 (2.80%)
Damage to the musculoskeletal system	Arthralgia, or pain in the joints, bone, jaw, back, neck, or chest	2	2 (1.40%)
Total		**143**	**100%**

#### 3.2.4 Evaluation of trigger utility

Our audit of the 375 patients who did not have a positive trigger revealed two patients who did not have a positive trigger but had an ADE. One patient had muscle soreness from suspected use of tadalafil and the other had headache from suspected use of selexipag tablets. The prevalence of false-negative triggering was 0.53% (2/375). The data for positive and negative cases and occurrence and non-occurrence of ADE are collated in Table 5. The sensitivity of the triggers was calculated to be 98.21% (110/112), the specificity was 72.57% (373/514), and the Correctness Index was 0.71.

**TABLE 5:** Statistical data of ADEs determined by the WHO-UMC method.

Trigger detection	Determination of ADEs by WHO-UMC evaluation method		Total
	ADEs	No ADEs	
Positive	110	141	251
Negative	2	373	375
Total	112	514	626

### 3.3 Analyses of risk factors for ADE occurrence

The results of the univariate statistical analysis in Table 2 showed that P < 0.05 for hospitalization duration, number of comorbidities, and number of positive triggers. Hence, these may be risk factors for ADE development in hospitalized patients with PAH. We conducted a multivariate binary logistic regression analysis with age, sex, previous hospitalizations, number of targeted drugs used concurrently, duration of hospital stay, number of comorbidities, and number of positive triggers as covariates, using ADE as the dependent variable.

The overall fit of the data to the binary logistic regression analysis model was 0.589. Hence, our data fitted well with the binary logistic regression model. A hospitalization duration of 11–20 days (odds ratio (OR) = 2.185), a number of comorbidities >9 (OR = 2.515), 2–4 positive triggers (OR = 10.110), and >4 positive triggers (OR = 17.382) all had P < 0.05 and OR > 1, suggesting that these factors were positively correlated with ADE occurrence and were risk factors for ADE occurrence (Table 6).

**TABLE 6:** Multifactor binary logistic regression analysis of ADEs.

Covariate	Categorization	β	OR	P
Sex	Male		1	
	Female	0.333	1.395	0.238
Age (years)	0–17		1	
	18–40	−0.291	0.747	0.632
	41–60	−0.612	0.542	0.304
	>60	−1.080	0.340	0.077
Duration of hospitalization (days)	1–10		1	
	**11**–**20**	0.782	**2.185**	**0.015**
	21–30	0.811	2.251	0.133
	>30	0.580	1.787	0.330
Number of comorbidities	1–3		1	
	4–6	0.523	1.687	0.130
	7–9	0.430	1.537	0.288
	**>9**	0.922	**2.515**	**0.045**
Number of targeted drug classes combined	1		1	
	2	0.400	1.491	0.185
	3	0.163	1.178	0.684
	>3	0.403	1.497	0.432
Number of positive triggers	0–1		1	
	**2**–**4**	2.314	**10.110**	**0.000**
	**>4**	2.855	**17.382**	**0.000**
Number of previous hospitalizations	1–3		1	
	4–6	0.090	1.094	0.781
	>6	0.085	1.089	0.829

Note: OR > 1, positive correlation; OR < 1, negative correlation; P < 0.05 is significant.

Note: Bold values indicate P < 0.05, suggesting that the change in this indicator has a significant correlation with the occurrence of ADEs and is a risk factor for the occurrence of ADEs.

According to the results of binary logistic regression analysis, the probability of ADE in patients with hospitalization duration of 11–20 days was 2.185-times higher than that in patients with hospitalization duration of 1–10 days. ADE prevalence in patients with >9 comorbidities was 2.515-times higher than that in patients with 7–9 comorbidities. ADE prevalence in patients with 2–4 positive triggers was 10.110-times higher than that in patients with 0–1 positive triggers. ADE prevalence in patients with >4 positive triggers was 17.382-times that in patients with 2–4 positive triggers.

### 3.4 Comparative analysis of spontaneous reporting and the GTT method

Among 626 patients with PAH treated with targeted drugs at the First Affiliated Hospital of Guangzhou Medical University between 1 January 2022 and 1 June 2023, the number of self-reported ADEs consisted of two cases involving two patients (0.32% (2/626)). The prevalence of detection of ADEs by the GTT method was 17.57%. We have organized the ADE data detected by the active monitoring and spontaneous reporting of the GTT method respectively into the following Table 7. The chi-square test revealed that the exact significance value (P value) was 0.00, which was less than 0.01, indicating that there was a significant difference in the results of the two monitoring methods. Therefore, compared with spontaneous reporting, the trigger monitoring method we have established can effectively improve the detection rate of ADEs.

**TABLE 7:** Detected ADE by spontaneous reporting and active monitoring by GTT method.

Detection of ADE		Spontaneous reporting		
		ADE occurred	No ADE occurred	Total
active monitoring by GTT method	ADE occurred	2	108	110
	No ADE occurred	0	516	516
	Total	2	624	626

The main reasons for the low spontaneous reporting rate of ADE in patients with PAH are the insufficient ability and skills of medical staff to identify ADE and concerns about responsibility. Doctors may be concerned about doctor-patient disputes and reputation damage caused by ADE reporting, as well as their unfamiliarity with the ADE reporting system and process, which leads to a relatively low enthusiasm of medical staff to report voluntarily. The GTT method established in this study can help medical staff quickly identify ADE and is conducive to improving the reporting rate of ADE.

## 4 Discussion

### 4.1 Discussion on bias factors

Whether the results of case review are correct or not is the main bias factor affecting the detection rate of ADE by the GTT method. To improve the accuracy of case review, we adopt the following measures: (1) Train the reviewers before the case review and organize them to study the training tutorial on ADE review in the GTT white paper. (2) The reviewers conduct the review independently. After the review is completed, the review results are compared. If any differences are found in the review results, they are handed over to two senior reviewers for re-examination to determine whether it is ADE. (3) When reviewing medical records, reviewers must strictly adhere to the “20-min principle” to ensure that the review time for each case is no less than 20 min. A study in the United States compared the internal review teams of hospitals with the hiring of experienced external teams and found that the sensitivity and specificity of the internal teams had both been checked by the experienced external teams (Sharek et al., 2011). In this study, a clinical pharmacist from the hospital was introduced as a junior reviewer, and a chief pharmacist and a chief physician were introduced as senior reviewers, effectively improving the sensitivity, specificity and detection rate of ADE of the trigger.

### 4.2 Effectiveness of trigger detection

Triggers are the basis for active monitoring of ADEs (Ji et al., 2018). We used a scientific method to extract and organize the initial triggers through several aspects, and we modified and improved the trigger items through a Delphi method. The overall PPV of the 24 triggers we established was 30.30%. According to previous studies, the overall PPV of triggers, in general, fluctuates between 10% and 40% (Magnéli et al., 2023). Hence, the overall PPV of the triggers established in our study was high.

In this real-world trial validating the clinical applicability of triggers, all 24 triggers were positive, and 22 triggers (91.67%) detected ADEs, of which 19 had an overall PPV >15%. L1 (absolute eosinophil value >0.3 × 10^9^/L) and S4 (flushing, facial flushing, hot flushes, rash, pruritus, urticaria, eczema) had high sensitivity, high specificity, and a PPV of 66.67%. Some triggers had a high prevalence of positive detection and a low prevalence of true-positives. An example of this phenomenon was S5 (peripheral edema, puffiness, edema, or swelling (including angioedema, lower-limb edema, fluid retention, retinal edema, and facial edema)), with 51 positive detections and only five true-positives, for a PPV of only 9.8%. This finding was considered to be due to pulmonary hypertension causing right-heart failure (Chinese Medical Association, Chinese Physicians Association, Pulmonary Vascular Disease Working Committee, 2021), which then leads to edema. Therefore, edema in some patients was caused by disease development rather than ADEs. A1 (use of antihypertensive drugs such as norepinephrine, dobutamine, mesoxamine, or ephedrine) is employed commonly for surgery or resuscitation, and less often to combat ADEs. Although Beraprost in S1 can increase the tendency of bleeding, including gastrointestinal bleeding, fundus bleeding, cerebral hemorrhage, etc., there are many interfering factors for bleeding in patients with PAH, making it difficult to determine the correlation between bleeding and medication, such as bleeding caused by excessive gastrointestinal ulcers in patients themselves. Theoretically, drug withdrawal due to ADE in T3 should be relatively common, such as withdrawal due to pulmonary fibrosis caused by Ambrisentan and withdrawal due to headache caused by Macitentan, etc. However, only two cases of ADE were detected in T3. The possible reason considered is that the number of included cases is relatively small. If the data volume is expanded, T3 may detect more ADE. For triggers with a relatively high false positive rate such as A1, S1, S5 and T3, it will increase the burden on reviewers when determining the “drug-ADE” association, but it will not lead to overtreatment because the prerequisite for intervening in patients is to determine that ADE has occurred, that is, to eliminate the interference of false positives.

T1 (transfer to ICU or higher) and S8 (renal injury, acute kidney injury, oliguria, anuria, acute tubular necrosis, tubular dysfunction, proteinuria, hematuria, chronic kidney damage) did not detect ADEs despite positive detection. A possible explanation for this observation is that during our review, patients with PAH were transferred to the ICU, more often than not, after surgery, usually unrelated to ADEs. Renal injury is usually judged using indicators that must be combined with serum urea nitrogen, serum creatinine, and urine output, and excluding renal impairment due to disease is important. However, considering that the amount of included case data is limited and the above two types of ADE are not included, if the data volume T1 and S8 is expanded, ADE will be detected. Therefore, two triggers are temporarily retained.

### 4.3 Characterization of ADEs

Of the 143 ADEs detected by a trigger, >80% of the drug–ADE associations were rated as “possibly relevant.” Possible explanations are that pulmonary hypertension is a rare disease and that patients often have multiple comorbidities simultaneously, so the occurrence of some ADEs can be explained by the effects of other diseases or other medications. Therefore, it is more rational to conclude that drug–ADE associations were “possibly relevant.” When encountering suspected ADEs in the clinic, the principle of “report when suspected” should be followed so that no ADEs are omitted.

According to the NCC MERP error-grading system, the ADEs detected in our study were predominantly “mild” and “moderate” (grades D, E, and F in 98.6% of cases). No ADEs were detected for permanent impairment or death (grades G and I). These observations may have been due to the small number of cases in our study and short timespan, which hampered the detection of long-term, rare, and unintended or severe ADEs. Ji et al. (2018) used triggers in the GTT method for active monitoring of ADEs in pediatric hospitalized patients. They detected ADE severity grades of E (82.6%), F (11.7%), and H (5.7%), which is consistent with the results of our study. Compared with spontaneous reporting for passive surveillance during the same time period, spontaneous reporting at the First Affiliated Hospital of Guangzhou Medical University identified only two E-level injuries, and no F- or H-level injuries were identified. Hence, the GTT method that we employed allowed for more comprehensive monitoring of ADEs.

ADEs after use of PAH-targeted drugs most frequently involved the hematologic system (46 cases, 32.17%), followed by the gastrointestinal system (38 cases, 26.57%), as well as the hepatic and renal systems (21 cases, 14.69%). The main manifestations were anemia, thrombocytopenia, leukopenia, nausea, diarrhea, increased serum urea nitrogen, abnormal creatinine level, and an abnormal ALT level. Therefore, it is recommended that physicians and nurses treating patients with PAH should pay attention to gastrointestinal symptoms as well as changes in levels of blood markers, ALT, AST, total bilirubin, serum creatinine, and serum urea nitrogen to prevent and cure ADEs in a timely manner.

### 4.4 Risk factors for ADE occurrence

Multifactorial binary logistic regression analyses showed that the risk factors for ADE occurrence in hospitalized patients with PAH included (but were not limited to) hospitalization duration of 11–20 days, number of comorbidities >9, 2–4 positive triggers, and >4 positive triggers.

In general, the longer the duration of hospitalization, the higher the prevalence of ADEs. Patients who stay longer in hospital usually require more treatments and interventions, and so are more likely to develop ADEs (Hwang et al., 2014). Tola et al. (2023) studied the incidence and factors influencing ADEs in pediatric cancer patients. They showed that 8 days of hospitalization was a risk factor for ADE occurrence.

We showed that the number of comorbidities >9 was a risk factor for ADE occurrence. Patients with more comorbidities are usually in poorer physical condition, have lower autoimmunity, and are less likely to tolerate the side effects of a medication and, thus, are more prone to ADE occurrence, than patients with fewer comorbidities. Sendekie et al. (2023) studied ADE incidence in adult patients at Gondar University General Specialized Hospital (Ethiopia). They found that the number of comorbidities had a significant correlation with ADE incidence, which is in line with the results of the present study.

## 5 Research shortcomings and outlook

First, this study is a single-center research and lacks multi-center external validation. Considering that different hospitals have different electronic medical record structures, the wide application of ADE automated detection in the hospital system requires adaptive optimization. Second, only 8 targeted drugs for the treatment of PAH were included, and no other commonly used drugs were included. There are certain limitations in the analysis. Third, unintended severe ADEs were not monitored, probably due to the small number of cases and short timespan. The successful identification of unintended severe ADEs usually requires long-term continuous follow-up and the exclusion of other intervening factors.

The ADE triggers for PAH inpatients constructed in this study, which have been embedded in the sample hospital’s HIS via CHPS, enable the real-time monitoring of ADEs, allowing for the continuous monitoring of long-term, chronic, cumulative adverse events, as well as that of unintended serious ADEs. Therefore, future studies will focus on monitoring unintended severe ADEs.

## 6 Conclusion

The triggers for ADEs in hospitalized patients with PAH established in our study based on the GTT method had high validity and practicability. These triggers could provide assistance for ADE monitoring in patients with PAH, improve the efficiency of ADE reporting, help better reflect the situation of ADEs, avoid the occurrence of predictable ADEs, improve the safety of medication administration in patients with PAH, and provide a reference for the improvement of clinical-treatment protocols. We explored one method of active monitoring of ADEs in patients with a rare disease (PAH). Our data could provide a reference for other similar diseases.

## Data Availability

The original contributions presented in the study are included in the article/supplementary material, further inquiries can be directed to the corresponding author.
